# Biocatalytic synthesis of flavones and hydroxyl-small molecules by recombinant *Escherichia coli* cells expressing the cyanobacterial *CYP110E1* gene

**DOI:** 10.1186/1475-2859-11-95

**Published:** 2012-07-18

**Authors:** Takuya Makino, Toshihiko Otomatsu, Kazutoshi Shindo, Emi Kitamura, Gerhard Sandmann, Hisashi Harada, Norihiko Misawa

**Affiliations:** 1 Research Institute for Bioresources and Biotechnology, Ishikawa Prefectural University, Suematsu, Nonoichi-shi, Ishikawa, 921-8836, Japan; 2 KNC Bio Research Center, KNC Laboratories Co., Ltd, 1-1-1, Murodani, Nishi-ku, Kobe, 651-2241, Japan; 3 Department of Food and Nutrition, Japan Women’s University, Mejirodai, Bunkyo-ku, Tokyo, 112-8681, Japan; 4 Molecular Biosciences 213, J. W. Goethe University, Max-von-Laue Str. 9, D-60054, Frankfurt/M, Germany; 5 Present address: Department of Biotechnology, Graduate School of Agricultural and Life Sciences, The University of Tokyo, Bunkyo-ku, Tokyo, 113-8657, Japan

**Keywords:** Cyanobacterium, Cytochrome P450, CYP110, Flavone synthase, Zerumbone, *Nostoc* sp. strain PCC 7120

## Abstract

**Background:**

Cyanobacteria possess several cytochrome P450s, but very little is known about their catalytic functions. *CYP110* genes unique to cyanaobacteria are widely distributed in heterocyst-forming cyanobacteria including nitrogen-fixing genera *Nostoc* and *Anabaena*. We screened the biocatalytic functions of all P450s from three cyanobacterial strains of genus *Nostoc* or *Anabaena* using a series of small molecules that contain flavonoids, sesquiterpenes, low-molecular-weight drugs, and other aromatic compounds.

**Results:**

*Escherichia coli* cells carrying each P450 gene that was inserted into the pRED vector, containing the RhFRed reductase domain sequence from *Rhodococcus* sp. NCIMB 9784 P450RhF (CYP116B2), were co-cultured with substrates and products were identified when bioconversion reactions proceeded. Consequently, CYP110E1 of *Nostoc* sp. strain PCC 7120, located in close proximity to the first branch point in the phylogenetic tree of the CYP110 family, was found to be promiscuous for the substrate range mediating the biotransformation of various small molecules. Naringenin and (hydroxyl) flavanones were respectively converted to apigenin and (hydroxyl) flavones, by functioning as a flavone synthase. Such an activity is reported for the first time in prokaryotic P450s. Additionally, CYP110E1 biotransformed the notable sesquiterpene zerumbone, anti-inflammatory drugs ibuprofen and flurbiprofen (methylester forms), and some aryl compounds such as 1-methoxy and 1-ethoxy naphthalene to produce hydroxylated compounds that are difficult to synthesize chemically, including novel compounds.

**Conclusion:**

We elucidated that the *CYP110E1* gene, C-terminally fused to the P450RhF RhFRed reductase domain sequence, is functionally expressed in *E. coli* to synthesize a robust monooxygenase, which shows promiscuous substrate specificity (affinity) for various small molecules, allowing the biosynthesis of not only flavones (from flavanones) but also a variety of hydroxyl-small molecules that may span pharmaceutical and nutraceutical industries.

## Background

**Figure 1:**
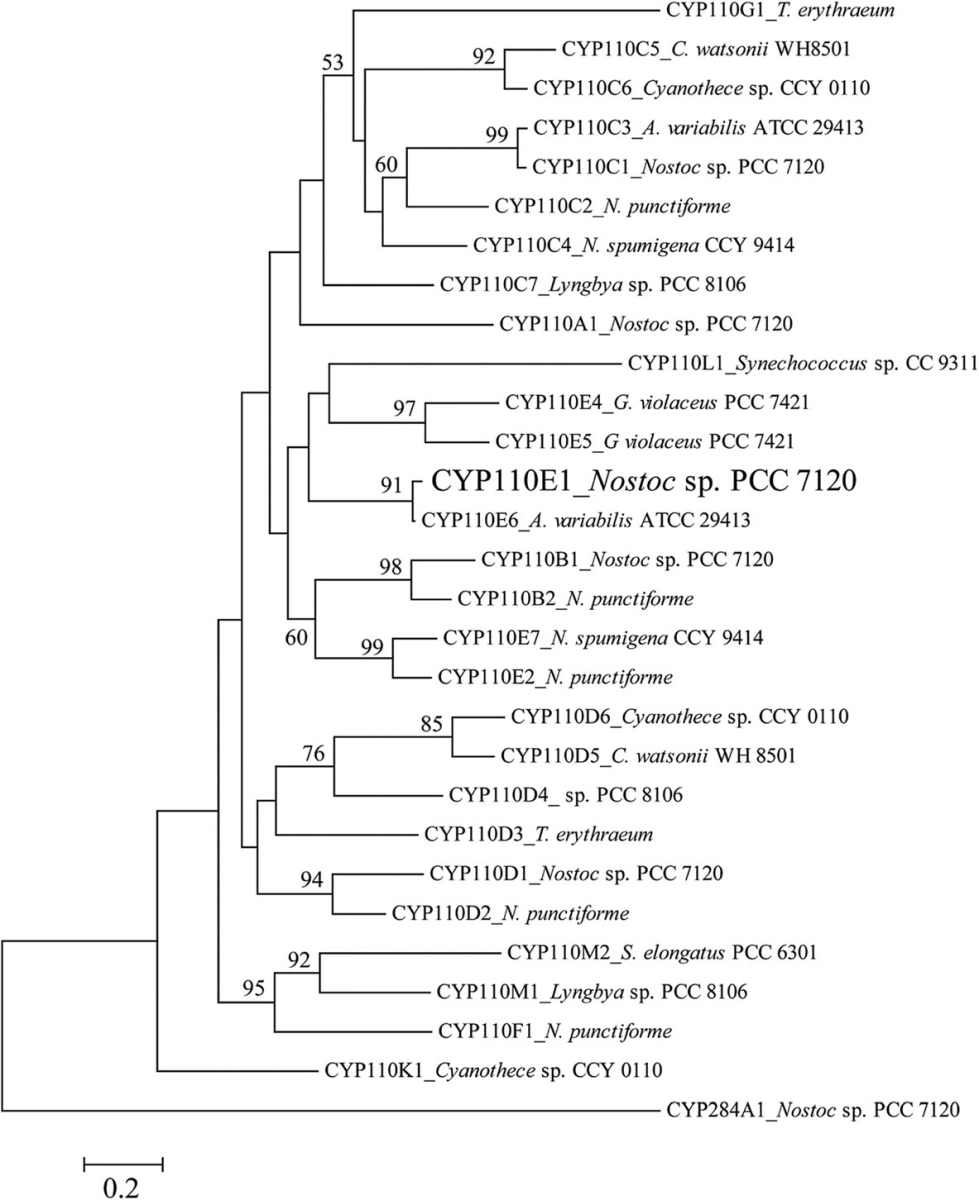
**Phylogenetic positions of cyanobacterial CYP110 family using 27 CYP110 proteins derived from 11 cyanobacteria, whose amino acid sequences are shown in CyanoBase.** The accession numbers in parentheses show those of respective P450 proteins. CYP110E1 is shown in boldface. The phylogenetic tree was constructed using the neighbor-joining method. The number shown next to each node indicates the percentage bootstrap value of 1,000 replicates (only 50% and higher are cited). The scale bar indicates a genetic distance of 0.02 (*Knuc*).

Cyanobacteria possess several cytochrome P450s (P450s), but only a few reports exist regarding their catalytic functions [1-3]. P450 CYP110 is a prominent family found in heterocyst-forming cyanobacteria including nitrogen-fixing genera *Nostoc* and *Anabaena*. *CYP110* genes are widely distributed in such cyanobacterial strains [1], e.g., *Nostoc* (also referred to as *Anabaena*) sp. strain PCC 7120 possesses five *CYP110* genes [classified as *CYP110A1* (alr1450), *CYP110B1* (all3746), *CYP110C1* (alr4686), *CYP110D1* (alr4766), and *CYP110E1* (alr4833)] in addition to one P450 gene of another family (*CYP284A1*) (Figure 1) [4]. The P450NS gene (*CYP110C1*) is positioned adjacent to the *NS1*gene encoding germacrene A synthase (alr4685) [2]. The encoded protein (CYP110C1) was recently confirmed to convert germacrene A to a hydroxylated sesquiterpene (1,2,3,5,6,7,8,8a-octahydro-6-isopropenyl-4,8a-dimethylnaphth-1-ol) [3]. *CYP110A1* is the first reported cyanobacterial P450 gene, which was present in a conserved 11.25-kb episomal element (the *nifD* element), and the encoded protein (CYP110A1) was hypothesized to be a fatty acid ω-hydroxylase, based on its substrate binding profile and amino acid sequence similarities to P450BM3 (CYP102A1) of *Bacillus megaterium* and the mammalian P450 family 4 fatty acid ω-hydroxylase [1,5].

In order to function as terminal monooxygenases, P450s must be associated with one or two additional proteins (or protein domains) to transfer two electrons from NAD(P)H to the heme domain of the P450 protein [6,7]. The vast majority of bacterial P450s need a FAD-containing ferredoxin reductase to receive electrons from NAD(P)H, and a ferredoxin (small iron-sulfur protein) to receive them from ferredoxin reductase, which subsequently reduces P450 itself [class I system; Additional file 1 Figure S1a[7]. P450RhF (CYP116B2) derived from *Rhodococcus* sp. NCIMB 9784 was discovered to be a self-sufficient P450 protein, in which the P450 domain is C-terminally fused to a reductase domain (here called RhFRed) [8]. RhFRed contained an FMN-binding ferredoxin reductase subdomain to receive electrons from NAD(P)H and a [2Fe-2S] ferredoxin subdomain [9]. A short linker region of 16 amino acids existed between P450 and RhFRed [8]. The redox chain of P450RhF resembles that of the class I system [Additional file 1 Figure S1c. Thus, vector pRED was constructed for the functional expression of bacterial P450 (class I) genes in *Escherichia coli*, using the linker sequence and RhFRed domain sequence [( Additional file 1Figure S2[10]. This vector has been demonstrated to be useful for functional expression of the P450cam gene (*CYP101A1*) [10,11], P450bzo gene (*CYP203A*) [10], P450balk gene (*CYP153A13a*) [12,13], other *CYP153A* genes [14], and P450 PikC gene [15], constituting corresponding self-sufficient P450 monooxygenation enzymes. We elucidate here that the *CYP110E1* gene is functionally expressed on pRED in *E. coli* to synthesize a robust monooxygenase, which shows promiscuous substrate specificity (affinity) for various small molecules.

## Results

### Screening experiments

*Nostoc* sp. strain PCC 7120, *Nostoc punctiforme* PCC 73102, and *Anabaena variabilis* ATCC 29413 possess six, ten, and four P450s, respectively. We screened the biocatalytic functions of these P450s using 47 small molecules that contain flavonoids, sesquiterpenes, low-molecular-weight drugs, naphthalene derivatives, and other chemicals with benzene rings [ Additional file 1 Figure S3)]. *E. coli* BL21 (DE3) cells carrying each P450 gene inserted into the pRED vector were co-cultured with the substrates and possible bioconversion products were analyzed by HPLC. Consequently, CYP110E1 of *Nostoc* PCC 7120 was found to be promiscuous for the substrate range mediating the biotransformation of various small molecules. The CYP110E1 enzyme that is C-terminally fused to RhFRed was confirmed to constitute the active P450 form by CO difference spectral analysis [ Additional file 1Figure S4]. Thus, cells of *E. coli* BL21 (DE3) carrying plasmid pCYP110E1-Red were used for the following experiments.

### Bioconversion of flavanones by *E. coli* (pCYP110E1-Red)

**Figure 2:**
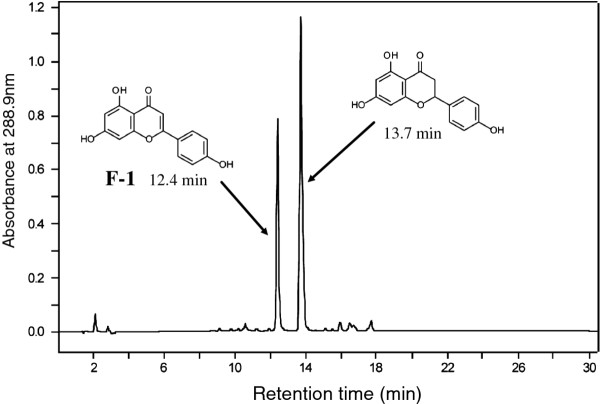
**HPLC analysis of the ethylacetate extract of the co-culture of cells of *E. coli*BL21 (DE3) carrying plasmid pCYP110E1-Red with naringenin.** 31.5% of naringenin was converted to **F-1**, which was identified as apigenin.

**Figure 3:**
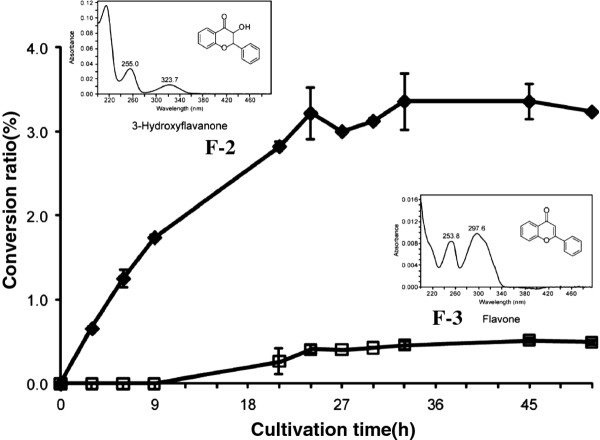
**Conversion rate of flavanone to F-2 [3-hydroxyflavanone (**♦**)] and F-3 [flavone (**□**)] by*E. coli*BL21 (DE3) cells carrying pCYP110E1-Red.** Error bars indicate the standard deviations obtained for three independent experiments. The conversion ratio (%) was measured by the ratio of peak area (max plot) in HPLC.

Naringenin was biotransformed to a product (**F-1**) with a conversion ratio of 31.5% (Figure 2) through co-cultivation with cells of *E. coli* (pCYP110E1-Red). **F-1** was identified as apigenin (4’,5,7-trihydroxyflavone) by its comparison with an authentic sample on HPLC analysis. Flavanone (RT 18.2 min) was converted to products **F-2** (RT 15.9 min; 5.7%) and **F-3** (RT 17.2 min; 2.1%), which were identified as 3-hydroxyflavanone and flavone, respectively, by their comparison with authentic samples on HPLC analysis. Figure 3 shows their production rate curves. Since this P450 was thought to biotransform various flavanones, we further examined 6-hydroxyflavanone (RT 15.9 min) and 7-hydroxyflavanone (RT 15.4 min). As a result, these hydroxyflavanones were converted to products **F-4** (RT 15.1 min; 12.1%) and **F-5** (RT 14.5 min; 1.4%), which were identified as 6-hydroxyflavone and 7-hydroxyflavone, respectively, by their comparison with authentic samples on HPLC analysis.

### Bioconversion of a sesquiterpene by *E. coli* (pCYP110E1-Red)

Only zerumbone among the examined terpenes [ Additional file 1: Figure S3] was biotransformed through co-cultivation with cells of *E. coli* (pCYP110E1-Red). The crude ethyl acetate (EtOAc) extract (152.0 mg) from this bioconversion mixture (200 ml), subjected to silica gel column chromatography (hexane-EtOAc = 2:1), yielded 5.6 mg (12.7%) of **S-1** (fr. 10–12), 3.2 mg (6.8%) of **S-2** (fr. 19–26), and 1.5 mg (3.2%) of **S-3** (fr. 30–40). These spectroscopic data are shown in Additional file 2.

**Figure 4:**
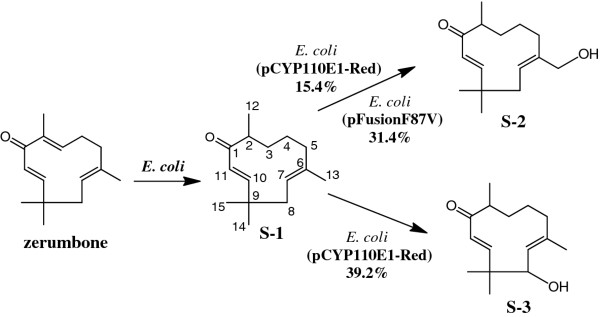
**Bioconversion of sesquiterpene zerumbone with *E. coli *BL21 (DE3) cells carrying pCYP110E1-Red.** % indicates conversion ratio measured by the ratio of peak area (max plot) in HPLC. *E. coli* BL21 (DE3) cells carrying plasmid pFusionF87V that expressed the P450BM3 (F87V) gene [17] were also examined for the ability to biotransform zerumbone, and the result is shown here.

The molecular formula of **S-1** was determined to be C_15_H_24_O (zerumbone + 2 H) by HREI-MS. Consistent with its molecular formula, **S-1** was proposed to be a product obtained by the reduction of a double bond in the substrate. The reduced double bond was determined to be ^2,3^Δ by the observation of a doublet methyl signal (δ_H_ 1.05, H-12), and the ^1^H-^13^C long range coupling from this doublet methyl to the ketone carbon (δ_C_ 205.1, C-1). The identity of **S-1** was thus determined as (6*E*,10*E*)-2,6,9,9-tetramethylcycloundeca-2,6-dien-1-one (Figure 4) [16]. Zerumbone was found to be converted to **S-1** with nontransformed *E. coli* BL21 (DE3) cells (data not shown). It was therefore thought that **S-2** and **S-3** were the genuine products by CYP110E1-Red.

The molecular formula of **S-2** was determined to be C_15_H_24_O_2_ by HREI-MS. Consistent with its molecular formula and ^1^H-NMR spectrum, the introduction of an alcoholic OH group in **S-1** was proposed. The position of the alcoholic OH group was clarified to be C-13 by the observation of an oxymethylene signal (δ_H_ 3.92 and δ_H_ 4.02, H-13) and the ^1^H-^13^C long range coupling from this oxymethylene to C-5 (δ_C_ 35.0), C-6 (δ_C_ 140.1), and C-7 (δ_C_ 126.0). The identity of **S-2** was thus determined as (6*Z*,10*E*)-6-hydroxymethyl-2,9,9-trimethylcycloundeca-2-ene-1-one (Figure 4). This product (**S-2**) was a novel compound according to the CAS database.

The molecular formula of **S-3** was determined to be C_15_H_24_O_2_ by HREI-MS. Consistent with its molecular formula and ^1^H-NMR spectrum, the introduction of an alcoholic OH group in **S-1** was proposed. The position of the alcoholic OH group was clarified to be C-8 by the observation of an oxymethylene signal (δ_H_ 4.24, H-8) and the ^1^H-^13^C long range coupling from H-14 (δ_H_ 1.12) and H-15 (δ_H_ 1.26) to C-8 (δ_C_ 75.5). The identity of **S-3** was thus determined as (6*E*,10*E*)-8-hydroxy-2,6,9,9-tetramethylcycloundeca-2,6-dien-1-one (Figure 4). This product (**S-3**) was a novel compound according to the CAS database.

### Bioconversion of aryl compounds by *E. coli* (pCYP110E1-Red)

A variety of aryl compounds, which include naphthalene derivatives and low-molecular-weight drugs, were biotransformed through co-cultivation with cells of *E. coli* (pCYP110E1-Red). Converted compounds were identified by chromatographic and spectroscopic analyses. Spectroscopic data are shown in Additional file 2.

#### Compounds converted from 1-methoxynaphthalene

The crude EtOAc extract (156.5 mg) from the bioconversion mixture (200 ml) with 1-methoxynaphthalene and *E. coli* (pCYP110E1-Red), subjected to silica gel column chromatography (hexane-EtOAc = 6:1), yielded 2.6 mg (7.5%) of **A-1** (fr. 13–15), 0.4 mg (1.1%) of **A-2** (fr. 19–22), and 0.7 mg (2.0%) of **A-3** (fr. 28–35).

**Figure 5:**
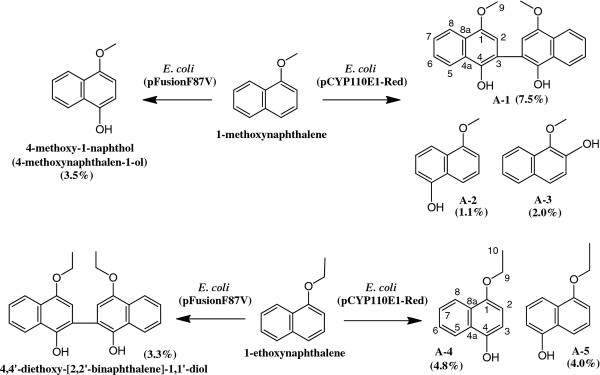
**Bioconversion of 1-methoxynaphthalene and 1-ethoxynaphthalene with *E. coli *BL21 (DE3) cells carrying pCYP110E1-Red.(**%) indicates yield (calculated from the weight of a purified product). The result with *E. coli* BL21 (DE3) cells carrying plasmid pFusionF87V [17] is also shown for comparison.

The molecular formula of **A-1** was determined to be C_22_H_18_O_4_ by HRAPCI-MS. Consistent with its molecular formula and ^1^H-NMR spectrum, **A-1** was proposed to be a secondary product (dimer) obtained by a phenol oxidation coupling reaction of the direct conversion product. The structure of A-1 was determined to be 4,4'-dimethoxy-[2,2'-binaphthalene]-1,1'-diol (Figure 5) by the observation of ^1^H-^13^C long range couplings from H-2 (2’) (δ 6.92) to C-1 (1’) (δ 149.3), C-3 (3’) (δ 120.4), and C-4 (4’) (δ 142.1), and ^1^H vicinal spin network of H-5 (5’) (δ 8.30) – H-6 (6’) (δ 7.54) – H-7 (7’) (δ 7.49) - H-8 (8’) (δ 8.20) [18].

The molecular formula of A-2 was determined to be C_11_H_10_O_2_ by HRAPCI-MS. Consistent with its molecular formula and ^1^H-NMR spectrum, the introduction of one phenolic OH group in the aromatic ring was proposed. The position of this phenolic OH group was determined to be C-5 by the observation of ^1^H-^1^H vicinal spin networks of H-2 (δ 6.84) – H-3 (δ 7.39) – H-4 (δ 7.74) and H-6 (δ 6.84) – H-7 (δ 7.30) – H-8 (δ 7.85), and an NOE observed between H-8 and H-9 (δ 3.99). The identity of A-2 was thus determined to be 5-methoxynaphthalen-1-ol (Figure 5) [17].

The molecular formula of A-3 was determined to be C_11_H_10_O_2_ by HRAPCI-MS. Consistent with its molecular formula and ^1^H-NMR spectrum, the introduction of one phenolic OH group in the aromatic ring was proposed. The position of this phenolic OH group was determined to be C-2 by the observation of ^1^H-^1^H vicinal spin coupling of H-3 (δ 7.23, d, *J* = 8.5 Hz) and H-4 (δ 7.57, d, *J* = 8.5 Hz) and ^1^H-^13^C long range couplings from H-4 to C-2 (δ 145.4) and C-5 (δ 128.3). The identity of A-3 was thus determined to be 1-methoxynaphthalen-2-ol (Figure 5) [19].

#### Compounds converted from 1-ethoxynaphthalene

The crude EtOAc extract (89.3 mg) from the bioconversion mixture (200 ml) with 1-ethoxynaphthalene and *E. coli* (pCYP110E1-Red), subjected to silica gel column chromatography (hexane-EtOAc = 6:1), yielded 1.8 mg (4.8%) of A-4 (fr. 10–12) and 1.5 mg (4.0%) of A-5 (fr. 14–16).

The molecular formula of A-4 was determined to be C_12_H_12_O_2_ by HRAPCI-MS. Consistent with its molecular formula and ^1^H-NMR spectrum, the introduction of one phenolic OH group in the aromatic ring was proposed. The position of this phenolic OH group was determined to be C-4 by the observation of ^1^H-^1^H vicinal spin coupling of H-2 (δ 7.55, d, *J* = 8.7 Hz) and H-3 (δ 7.23, d, *J* = 8.7 Hz) and the ^1^H-^13^C long range couplings from H-5 (δ 7.78) to C-4 (δ 145.8). The identity of **A-4** was thus determined to be 4-ethoxynaphthalen-1-ol (Figure 5) [20].

The molecular formula of **A-5** was determined to be C_12_H_12_O_2_ by HRAPCI-MS. Consistent with its molecular formula and ^1^H-NMR spectrum, the introduction of one phenolic OH group in the aromatic ring was proposed. The position of this phenolic OH group was determined to be C-5 by the observation of ^1^H-^1^H vicinal spin networks of H-2 (δ 6.83) – H-3 (δ 7.37) – H-4 (δ 7.71), and H-6 (δ 6.85) – H-7 (δ 7.29) – H-8 (δ 7.89), and the ^1^H-^13^C long range couplings from H-3 to C-1 (δ 154.8) and C-4a (δ 125.4) and from H-7 to C-5 (δ 151.2) and C-8a (δ 127.3). The identity of **A-5** was thus determined to be 5-ethoxynaphthalen-1-ol (Figure 5) [21].

#### Compounds converted from 2-methylnaphthalene

The crude EtOAc extract (175.0 mg) from the bioconversion mixture (200 ml) with 2-methylnaphthalene and *E. coli* (pCYP110E1-Red), subjected to silica gel column chromatography (hexane-EtOAc = 5:1), yielded 1.2 mg (3.8%) of **A-6** (fr. 11–13) and 1.5 mg (4.7%) of **A-7** (fr. 20–24).

**Figure 6:**
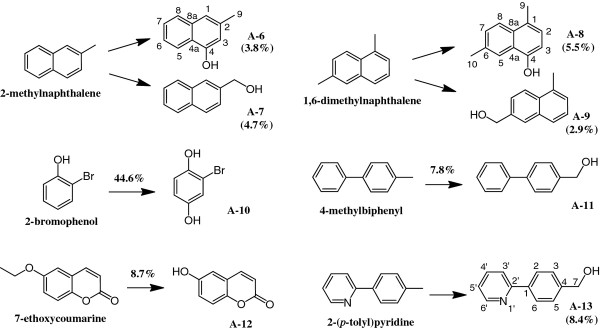
**Bioconversion of other aryl compounds with *E. coli *BL21 (DE3) cells carrying pCYP110E1-Red.** % and (%) indicate conversion ratio and yield, respectively.

The molecular formula of **A-6** was determined to be C_11_H_10_O by HRAPCI-MS. Consistent with its molecular formula and ^1^H-NMR spectrum, the introduction of one phenolic OH group in the aromatic ring was proposed. The position of this phenolic OH group was determined to be C-4 by the observation of two singlet sp^2^ methines (δ 6.67 and δ 7.22) and the ^1^H-^1^H vicinal spin networks of H-5 (δ 8.10) – H-6 (δ 7.40) – H-7 (δ 7.43) - H-8 (δ 7.71). The identity of **A-6** was thus determined to be 3-methylnaphthalen-1-ol (Figure 6). **A-7** was identified as naphthalene-2-ylmethanol (Figure 6) with HPLC analysis by its comparison with an authentic sample extracted from co-culture with 2-methylnaphthalene and *E. coli* BL21 cells carrying plasmid pUCRED-Balk, which expressed the *CYP153A13a* gene [13].

#### Compounds converted from 1,6-dimethylnaphthalene

The crude EtOAc extract (95.7 mg) from the bioconversion mixture (200 ml) with 1,6-dimethylnaphthalene and *E. coli* (pCYP110E1-Red), subjected to silica gel column chromatography (hexane-EtOAc = 6:1), yielded 1.9 mg (5.5%) of **A-8** (fr. 12–15) and 1.0 mg (2.9%) of **A-9** (fr. 21–26).

The molecular formula of **A-8** was determined to be C_12_H_12_O by HRAPCI-MS. Consistent with its molecular formula and ^1^H-NMR spectrum, the introduction of one phenolic OH group in the aromatic ring was proposed. The position of this phenolic OH group was determined to be C-4 by the observation of ^1^H-^1^H vicinal spin couplings of H-2 (δ 7.05, d, *J* = 7.9 Hz) and H-3 (δ 6.70, d, *J* = 7.9 Hz), and H-7 (δ 7.37, d, *J* = 8.6 Hz) and H-8 (δ 7.84, d, *J* = 8.6 Hz), and ^1^H-^13^C long range couplings from H-9 (δ 2.58) to C-1 (δ 126.6), C-2 (δ 125.1), and C-8a (δ 131.7). The identity of **A-8** was thus determined to be 4,7-dimethylnaphthalen-1-ol [17]. **A-9** was identified as (5-methylnaphthalen-2-yl)methanol (Figure 6) with HPLC analysis by its comparison with an authentic sample extracted from co-culture with 1,6-dimethylnaphthalene and *E. coli* BL21 (pUCRED-Balk) [13].

##### A compound converted from 2-bromophenol

The EtOAc extract from the bioconversion mixture (0.5 ml) with 2-bromophenol and *E. coli* (pCYP110E1-Red) was subjected to HPLC to yield a product (**A-10**). **A-10** was identified as 2-bromobenzene-1,4-diol (Figure 6) with HPLC by its comparison with an authentic sample extracted from co-culture with 2-bromophenol and *E. coli* BL21 (pUCRED-Balk) [13].

##### A compound converted from 4-methylbiphenyl

The EtOAc extract from the bioconversion mixture (0.5 ml) with 4-methylbiphenyl and *E. coli* (pCYP110E1-Red) was subjected to HPLC to yield a product (**A-11**). **A-11** was identified as [1,1’-biphenyl]-4-ylmethanol (Figure 6) with HPLC by its comparison with an authentic sample extracted from co-culture with 4-methylbiphenyl and *E. coli* BL21 (pUCRED-Balk) [13].

##### A compound converted from 7-ethoxycoumarine

The EtOAc extract from the bioconversion mixture (0.5 ml) with 7-ethoxycoumarine and *E. coli* (pCYP110E1-Red) was subjected to HPLC to yield a product (**A-12**). **A-12** was identified as 6-hydroxy-2 H-chromen-2-one (Figure 6) with HPLC by its comparison with an authentic sample.

##### A compound converted from 2-(*p*-tolyl)pyridine

The crude EtOAc extract (103.7 mg) from the bioconversion mixture (200 ml) with 2-(*p*-tolyl)pyridine and *E. coli* (pCYP110E1-Red), subjected to silica gel column chromatography (CH_2_Cl_2_-MeOH = 20:1), yielded 3.1 mg (8.4%) of **A-13** (fr. 23–27). The molecular formula of **A-13** was determined to be C_12_H_11_NO by HREI-MS. The ^1^H- and ^13^C-NMR spectra showed the methyl group in the substrate was oxidized to the corresponding primary alcohol. The identity of A-13 was thus determined to be (4-(pyridin-2-yl)phenyl)methanol (Figure 6), which was also produced through co-culture with 2-(*p*-tolyl)pyridine and *E. coli* BL21 (pUCRED-Balk) [13].

##### A compound converted from ibuprofen methylester

**Figure 7:**
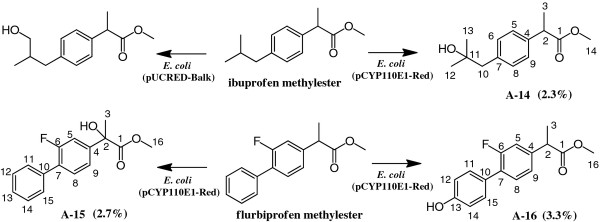
**Bioconversion of drugs ibuprofen methylester and flurbiprofen methylester with*E. coli*BL21 (DE3) cells carrying pCYP110E1-Red.** (%) indicates yield. The result with *E. coli* BL21 cells carrying plasmid pUCRED-Balk that expressed the P450balk gene (*CYP153A13a*) from an alkane-assimilating marine bacterium *Alcanivorax borkumensis*[13] is also shown for comparison.

The crude EtOAc extract (225.0 mg) from the bioconversion mixture (200 ml) with ibuprofen methylester and *E. coli* (pCYP110E1-Red), subjected to silica gel column chromatography (hexane-EtOAc = 4:1), yielded 1.1 mg (2.3%) of **A-14**. The molecular formula of **A-14** was determined to be C_14_H_20_O_3_ by HREI-MS. Consistent with its molecular formula and ^1^H-NMR, the introduction of one alcoholic OH group in the substrate was proposed. The position of this alcoholic OH group was determined to be C-11 because all signals of H-10, H-12, and H-13 were observed to be singlet. The identity of **A-14** was thus determined to be methyl 2-(4-(2-hydroxy-2-methylpropyl)phenyl)propanoate (Figure 7) [22].

#### Compounds converted from flurbiprofen methylester

The crude EtOAc extract (254.7 mg) from the bioconversion mixture (200 ml) with flurbiprofen methylester and *E. coli* (pCYP110E1-Red), subjected to silica gel column chromatography (hexane-EtOAc = 4:1), yielded 1.5 mg (2.7%) of **A-15** (fr 10–12) and 1.8 mg (3.3%) of **A-16** (fr 23–28).

The molecular formula of **A-15** was determined to be C_16_H_15_FO_3_ by HREI-MS. Consistent with its molecular formula and ^1^H-NMR spectrum, the introduction of one alcoholic OH group in the substrate was proposed. The position of this alcoholic OH group was determined to be C-2 because the signal of H-3 (δ 1.80) was observed to be singlet. The identity of **A-15** was thus determined to be methyl 2-(2-fluoro-[1,1'-biphenyl]-4-yl)-2-hydroxypropanoate (Figure 7).

The molecular formula of **A-16** was determined to be C_16_H_15_FO_3_ by HRAPCI-MS. Consistent with its molecular formula and ^1^H-NMR spectrum, the introduction of one phenolic OH group in the aromatic ring was proposed. The position of this phenolic OH group was determined to be C-13 because the signals of H-12 and H-14 were observed to be doublet (*J* = 8.6 Hz) and at high field (δ 6.89). The identity of **A-16** was thus determined to be methyl 2-(2-fluoro-4'-hydroxy-[1,1'-biphenyl]-4-yl)propanoate (Figure 7).

## Discussion

The phylogenetic analysis (Figure 1) showed that CYP110E1 of *Nostoc* sp. strain PCC 7120 was located in close proximity to the first branch point in the phylogenetic tree of the CYP110 family, *i.e*., among 27 CYP110 proteins derived from 11 cyanobacteria, only four P450s, CYP110K1, CYP110D2, CYP110E6, and CYP110D3, were located closer to the first branch point than CYP110E1. In this study, CYP110E1, whose function had been unknown, was found to function as a substrate-promiscuous monooxygenase when it was C-terminally fused to the RhFRed reductase domain of P450RhF (CYP116B2) by the use of the pRED vector. Naringenin was converted directly to apigenin with a significant conversion ratio (31.5%) (Figure 2). Naringenin and apigenin belong to typical flavonoids that can be biosynthesized in higher plants. Artificial flavonoids, flavanone, 6-hydroxyflavanone and 7-hydroxyflavanone, were also converted to the respective flavones, even if with low conversion ratios, *i.e*., major parts of the substrates remained without being biotransformed even after 48-h co-culture. These results revealed that CYP110E1 functions as a flavone synthase. Such an activity is reported for the first time in prokaryotic P450s. When using flavanone as the substrate, 3-hydroxyflavanone was additionally generated (Figure 3). It may be possible that 3-hydroxyflavanone is the intermediate from flavanone to flavone. However, *E. coli* (pCYP110E1-Red) did not biotransform 3-hydroxyflavanone when it was added as the substrate (data not shown). It is therefore likely that flavones and 3-hydroxyflavanone were generated independently from flavanone. The higher-plant CYP93B was characterized as P450-type flavone synthase (FSII) and was proposed to convert flavanones to flavones by way of 2-hydroxyflavanones [23,24]. Such a catalytic route may be the case with CYP110E1.

P450BM3 (also described as P450BM-3 or P450_BM3_; CYP102A1) derived from *Bacillus megaterium*, one of the best-characterized prokaryotic P450s, is a natural fusion enzyme composed of a P450 part and a eukaryote-type NADPH-P450 reductase domain Additional file 1: Figure 1d[25-27]. This P450 part is closely related to the CYP110 family [1] and exhibited 24.6% amino acid sequence identity to CYP110E1. P450BM3, whose native substrates are thought to be long-chain fatty acids, has been shown to possess substrate and catalytic promiscuities [28-31]. Specifically, P450BM3 variants incorporating active site mutations that include F87V (or F87A) were found to acquire broader substrate affinity not only for a variety of aryl compounds including substituted naphthalenes [17,32,33], but also for the monoterpene α-pinene and the sesquiterpene amorpha-4,11-diene [30,34]. The P450BM3 variant F87V that was N-terminally fused to an archaeal peptidyl-prolyl *cistrans* isomerase (PPIase), which was synthesized by the plasmid named pFusionF87V in *E. coli* cells, was shown to elevate the stability of the P450 protein [17].

Zerumbone, a sesquiterpene contained in the shampoo ginger (*Zingiber zerumbet* Smith), is a promising chemopreventive agent, since its anti-inflammatory and anti-tumor activities have been investigated [35-38]. *E. coli* BL21 (DE3) carrying plasmid pCYP110E1-Red was shown to hydroxylate zerumbone to produce two novel compounds (**S-2** and **S-3**) via the endogenous metabolite in *E. coli* (**S-1**; Figure 4). On the other hand, *E. coli* BL21 (DE3) carrying plasmid pFusionF87V was not able to synthesize compound **S-3** from zerumbone, although it bioconverted zerumbone to compound **S-2** with a higher conversion ratio than that of *E. coli* (pCYP110E1-Red) (Figure 4). *E. coli* (pFusionF87V) was able to hydroxylate β-eudesmol (a sesquiterpene contained in edible plants of the *Zingiberaceae* family) at its C-5 position [17], while *E. coli* (pCYP110E1-Red) was not able to biotransform β-eudesmol (data not shown). The two prokaryotic P450s, CYP110E1 and P450BM3 (F87V), are likely to have the ability to biotransform some sesquiterpenes of higher-plant origin complementally. CYP109B1 from *Bacillus subtilis* was also found to possess a wide substrate range for saturated fatty acids, *n*-alcohol, and some isoprenoids, and convert the sesquiterpene (+)-valencene to (+)-nootkatone, a high added-value compound found in grapefruit juice [39,40].

*E. coli* (pCYP110E1-Red) was found to biotransform various aryl compounds. 1-Methoxynaphthalene and 1-ethoxynaphthalene were converted to several hydroxylated compounds (Figure 5). When using 1-methoxynaphthalene as the substrate, an aryl coupling reaction was observed to produce compound **A-1**. This product is likely to be generated via oxidative aryl coupling through a non-enzymatic dimerization process from 4-methoxynaphthalen-1-ol, which is speculated to be the direct enzyme product. Misawa et al. (2011) showed that these two substrates were biotransformed by *E. coli* (pFusionF87V) to hydroxylated compounds different from those obtained using *E. coli* (pCYP110E1-Red) (Figure 5) [17]. In this case, oxidative aryl coupling was observed in the reaction, not from 1-methoxynaphthalene but from 1-ethoxynaphthalene to produce 4,4'-diethoxy-[2,2'-binaphthalene]-1,1'-diol [17]. These findings suggest that the two P450s, CYP110E1 and P450BM3 (F87V), are useful for producing a variety of 1-methoxy and 1-ethoxy naphthalene derivatives, whose naphthalene rings acquire one hydroxyl group without eliminating the methyl or ethyl group. Such an elimination is often likely to occur, e.g., CYP153A13a (P450balk) from *Alcanivorax borkumensis* SK2, which was found to possess a promiscuous substrate range for aryl compounds and aromatic compounds including alkyl groups, converted 1-methoxy naphthalene to 1-naphthol [12,13]. Figure 6 shows other aryl compounds biotransformed with *E. coli* (pCYP110E1-Red). The reactions towards products **A-7A-9A-10A-11**, and **A-13** were observed also in *E. coli* BL21 (pUCRED-Balk) that expressed the *CYP153A13a* gene [13]. On the other hand, *E. coli* (pFusionF87V) converted 2-methylnaphthalene to compounds 2-methyl-1-naphthol and 6-methy-1-naphthol, which are different from those with *E. coli* (pCYP110E1-Red), while it converted 1,6-dimethylnaphthalene to the same compound **A-8**[17].

The present study also showed that *E. coli* (pCYP110E1-Red) biotransformed the methylester forms of non-steroidal anti-inflammatory drugs ibuprofen and flurbiprofen to produce the respective hydroxyl derivatives that are difficult to synthesize chemically (Figure 7). *E. coli* (pUCRED-Balk) converted ibuprofen methylester to another hydroxyl substituent [13], while it was not able to biotransform flurbiprofen methylester (data not shown). The four drug metabolites produced by recombinant *E. coli* cells (Figure 7) or their free carboxylate forms, awaiting the determination of the absolute configuration, could be used as standards in studies on the metabolisms of ibuprofen and flurbiprofen with human P450s [41].

## Conclusion

The present study revealed that cyanobacterial cytochrome P450 CYP110E1, C-terminally fused to the P450RhF (CYP116B2) RhFRed reductase domain, is promiscuous for substrate and catalytic ranges and is useful for biosynthesizing not only flavones (from flavanones), but also a variety of hydroxyl- small molecules that are difficult to synthesize chemically, which may span pharmaceutical and nutraceutical industries.

## Methods

### Bacterial strains and genetic manipulation

Three cyanobacterial strains, *Nostoc* sp. strain PCC 7120, *Nostoc punctiforme* PCC 73102 (=ATCC 29133), and *Anabaena variabilis* ATCC 29413 were obtained from Pasteur Culture collection, Paris, and grown autotrophically in BG 11 medium. For the isolation of genomic DNA, cyanobacteria were harvested from the log phase and were immediately treated with lysozyme (10 mg/ml for 1 h). Genomic DNA was then isolated with the GenElute plant genomic DNA kit (Sigma-Aldrich, St. Louis, MO).

*E. coli* DH5α (*ECOS* Competent *E. coli* DH5α; Nippon Gene, Tokyo, Japan) was utilized as the host for DNA manipulations. *E. coli* BL21 (DE3) (Nippon Gene) was used for the functional expression of each P450 gene, which was inserted into the pRED vector [10]. PCR amplifications were performed using Prime STAR Max Premix DNA polymerase (Takara Bio, Ohtsu, Japan) and a thermal cycler (Applied Biosystems, Foster City, CA). Restriction enzymes and DNA-modifying enzymes were purchased from New England BioLabs (Beverly, CA) or Takara Bio. A Ligation-Convenience Kit (Nippon Gene) was also used. Plasmid DNA was prepared with a QIAprep Miniprep Kit (Qiagen, Hilden, Germany). All recombinant DNA experiments were carried out according to the suppliers’ manuals or Sambrook and Russell (2001) [42].

### Nucleotide sequencing and computer analysis

Nucleotide sequences were confirmed with Bigdye terminator cycle sequencing ready reaction kit version 3.1 (Applied Biosystems) and a model 3730 DNA analyzer (Applied Biosystems). Homologous protein sequences in the protein sequence database were retrieved from CyanoBase of Kazusa DNA Research Institute (http://genome.kazusa.or.jp/cyanobase) with the BLAST program [43], and aligned by Clustal W program in Molecular Evolutionary Genetics Analysis (MEGA) software version 5.0 (http://www.megasoftware.net/). A phylogenetic tree was also constructed according to MEGA 5.0.

### Construction of plasmids

Cyanobacterial P450 genes were amplified by PCR from genomic DNA of *Nostoc* sp. PCC 7120, *N. punctiforme* PCC 73102, or *A. variabilis* ATCC 29413. All synthetic oligonucleotides used in this work were listed in Table 1. PCR amplification was performed in a 50 μl reaction mixture containing 25 ng of genomic DNA, 25 μl of 2 × the DNA polymerase, 10 μM of each primer, and 5% dimethyl sulfoxide (DMSO). The PCR conditions used were the following: preincubation at 98 °C for 2 min; a total of 5 cycles at 98 °C for 10 sec, 55 °C for 10 sec, and 75 °C for 15 sec; a total of 30 cycles at 98 °C for 10 sec, 62 °C for 5 sec and 75 °C for 15 sec. An amplified 1.4 kb fragment was digested with *Nde*I and *Eco*RI or *Hin*dIII, and ligated into the *Nde*I-*Eco*RI or *Nde*I-*Hin*dIII site of pRED to construct the desired plasmids. In these plasmids, the stop codons of the respective P450 genes were removed to fuse the N-terminus of RhFRed.

**Table 1:** List of primers used in this study

	**primer**	**sequence**	**species**
CYP284A1	Ana1361F	5'-TAC CAT ATG ATG CTC CAA TAC ATT ACT GCT CTC-3'	
	Ana1361R	5'-TAC GAA TTC ATT TCT CAA CCG AAA GCG CAC T-3'	
CYP110A1	Ana1450F	5'-TAC CAT ATG ATG TTG ACT CAA TTA CCA AAT CC-3'	
	Ana1450R	5'-TAC GAA TTC GTT GAA AAT CTT GCT ACT TTG CT-3'	
CYP110B1	Ana3746F	5'-TAC CAT ATG ATG CAC CTA CCA AAA GG-3'	
	Ana3746R	5'-CAC GAA TTC ACT TAC AGT AGT TGT TTC TAG-3'	
			***Nostoc*sp. PCC 7120**
CYP110C1	Ana4686F	5'-CAC CAT ATG ATG AAA TAT CAA ATA CAG AGA CC-3'	
	Ana4686R	5'-TAC AAG CTT TGC GTT GAA TGT TGT TGA G-3'	
CYP110D1	Ana4766F	5'-TAC CAT ATG ATG ACA GTC ACT CAA AAC C-3'	
	Ana4766R	5'-CAC AAG CTT CGA ATT ACG CAT TCT TTT ATT AG-3'	
CYP110E1	Ana4833F	5'-TAC CAT ATG ATG AAA CTT CCA GAT AGT C-3'	
	Ana4833R	5'-TAC GAA TTC TAC TTC TAC AGG GTT TTT G-3'	
CYP110C3	Ana1981F	5'-TAC CAT ATG ATG AAG TAT CAA ATA AAG AGA C-3'	
	Ana1981R	5'-TAC GAA TTC TGT TGT GAA TGT TGT TGA G-3'	
CYP110E6	Ana2103F	5'-TAC CAT ATG ATG AAA CTT CCA GAT AGT CC-3'	
	Ana2103R	5'-TAC GAA TTC TAC TTC TAC AGG GCT TTT GA-3'	
			***A. variabilis*ATCC 29413**
CYP110A2	Ana3921F	5'-TAC CAT ATG ATG TTG ACT CAA TTA CCA AA-3'	
	Ana3921R	5'-CAC GAA TTC ATT AAA AAT CTT GTT ACT TTG CT-3'	
CYP284A3	Ana4063F	5'-TAC CAT ATG ATG CTC CAA TAC GTT ACT GCT C-3'	
	Ana4063R	5'-TAC GAA TTC ATT TCT CAA CCG AAA GCG CAC-3'	
CYP110F1	Nos0984F	5'-TAC CAT ATG ATG AAA ATA CTT GAT AGT CTA AC-3'	
	Nos0984R	5'-TAC AAG CTT AGT AGA AAG TAT TGT TTG TCT TT-3'	
CYP110B2	Nos0985F	5'-TAC CAT ATG ATG AAA TTA CCA AAA GGC C-3'	
	Nos0985R	5'-TAT GAA TTC AAC AGT GGC TGT CTG-3'	
CYP197B1	Nos2212F	5'-TAC CAT ATG ATG GTT GCC GAT GTA TT-3'	
		5'-CAC GAA TTC TTT AGA AGT GTC TAA TGC AA-3'	
CYP284A2	Nos2399F	5'-TAC CAT ATG ATG TTC CAA CAG ATT GCT GC-3'	
	Nos2399R	5'-TAC GAA TTC ACG AGC GAT ATT GTC AGA GT-3'	
CYP120B1	Nos2686F	5'-TAC CAT ATG ATG AAA ACT AAT CAA ATT CCT-3'	
	Nos2686R	5'-TAT GAA TTC CCG AGG TTG AAA TCT-3'	
			***N. punctiforme*PCC 73102**
CYP110D2	Nos3640F	5'-CTA C CA TAT GAT GAA AAG TCG TAA CAA TAA AA-3'	
	Nos3640R	5'-TAT GAA TTC AAC TAG GGC TGG C-3'	
CYP110C2	Nos6291F	5'-TAC CAT ATG ATG CAA CTA CCT AAT ATT CT-3'	
	Nos6291R	5'-TAT GAA TTC GGA TAG GGG TGT AG-3'	
CYP110E2	Nos7017F	5'-GCA GCA TAT GAT GTC TTT ACT TAA ACT G-3'	
	Nos7017R	5'-TCA C GA ATT CAA CTG AAC TAG AGC T-3'	
CYP227A1	Nos7684F	5'-ATA C CA TAT GAT GAC ACT TAA AGA TAA AG-3'	
	Nos7684R	5'-TAT GAA TTC CAG TCG TTG AGC AA-3'	
CYP120C1	Nos8095F	5'-TAC CAT ATG ATG CAG CAG TTA AAA TCC G-3'	
	Nos8095R	5'-TAC GAA TTC ACT ATC CAA GGG ATG CTT T-3	

### CO difference spectral analysis

CO difference spectral analysis was done as described [12].

### Bioconversion experiments

*E. coli* BL21 (DE3) carrying each plasmid was grown in an LB medium including ampicillin (Ap; 100 μg/ml) at 37°C with rotary shaking for 3–4 h until the absorbance at OD 600 nm reached approximately 0.8. For screening experiments, 1.5 ml of this preculture was inoculated into 125 ml of an LB medium including Ap (100 μg/ml), 5-aminolevulinic acid hydrochloride (5-ALA; 80 μg/ml), ammonium iron (II) sulfate (0.1 mM), and IPTG (0.1 mM) in a baffled Erlenmeyer flask, and cultured at 20°C for 20 h on a rotary shaker (140 rpm; Kuhner Shaker Lab Therm LT-X, Basel, Switzerland). Cells were collected by centrifugation and resuspended in 25 ml of CV-3 buffer [sodium phosphate buffer (50 mM, pH 7.2) containing 5% glycerol] in a baffled Erlenmeyer flask. Five hundred μl of this cell suspension was added into each well of a 96 well sterile plate (PR-Master Block 2ML; Greiner Bio-One, Frickenhausen, Germany), together with 1 mM (final concentration) of substrate dissolved in dimethyl sulfoxide (DMSO). Bioconversion was performed by cultivation at 25°C for 24 h with 300 rpm using the Kuhner Shaker.

For structural determination of products, large scale cultivation was carry out, by inoculation of 5 ml of the preculture into 500 ml of LB medium including Ap (100 μg/ml), 5-ALA (80 μg/ml), ammonium iron (II) sulfate (0.1 mM), and IPTG (0.1 mM) in a baffled Erlenmeyer flask at 20°C for 20 h with 140 rpm on the Kuhner Shaker. Cells were collected by centrifugation, and resuspended in 100 ml of CV-3 buffer in a baffled Erlenmeyer flask. Each substrate dissolved in DMSO was added at a final concentration of 1 mM to the cell suspension and bioconversion was performed by cultivation at 25°C for 48 h with 180 rpm.

Substrates and authentic samples used in this study were purchased from Tokyo Chemical Industry Co. (Tokyo, Japan), Sigma-Aldrich Co. (St. Louis, MO), or Wako Pure Chemical Industries (Osaka, Japan).

### Chemical synthesis of ibuprofen methylester and flurbiprofen methylester

Zero point six mol/l of trimethylsilyldiazomethane in hexane (12.5 ml, 7.5 mmol; Tokyo Chemical Industry) was added, drop by drop on ice, to a solution (33 ml, benzene/methanol = 2/1) containing ibuprofen (1.03 g, 5 mmol; Wako Pure Chemical Industries) or flurbiprofen (1.22 g, 5 mmol; Wako Pure Chemical Industries). The reaction mixtures from ibuprofen and flurbiprofen were stirred at room temperature for 5 h. After concentrated to dryness, the respective residues (1.23 g and 1.53 g) were subjected to column chromatography (I.D. 20 mm × 300 mm) with silica gel IR-60-63/210-w (Daiso Co., Osaka Japan), developed in hexane-ethyl acetate (EtOAc) = 2:1 and hexane-EtOAc = 10:1, to yield 0.79 g of ibuprofen methylester as a colorless oil and 1.27 g of flurbiprofen methylester as a white powder.

### Extraction and HPLC analysis of products

Five hundred μl of the reaction mixture liquid was added to 100 μl of saturated sodium chloride solution and 500 μl of EtOAc and shaken for 5 min. After centrifugation, the organic phase was analyzed by high pressure liquid chromatography (HPLC; Waters 2695, Waters Corp., Milford, MA) equipped with an on-line photodiode array detector (Waters 2996). An aliquot of the organic phase (20 μl) was applied to HPLC and separated using an XTerra MS C_18_ column (I.D. 4.6 mm × 100 mm; Waters), and a flow rate of 1 ml/min was used, with solvent A [5% acetonitrile (CH_3_CN) in 20 mM phosphoric acid] for 3 min, then a linear gradient from solvent A to solvent B (95% CH_3_CN in 20 mM phosphoric acid) for 25 min, and finally with solvent B for 15 min, with the maximum absorbance being monitored in the range of 200–500 nm (max plot).

### Purification and identification of products

The liquid phase containing the reaction mixture (200 ml; 100 ml x 2) was extracted with EtOAc (200 ml x 2 times). The resultant organic layer was concentrated *in vacuo* and analyzed by thin-layer chromatography (TLC) on silica gel (E. Merck 60 F-254 0.25-mm silica gel plates). Products were purified by column chromatography on Silica Gel 60 [20 mm (diameter) x 250 mm (length); Merck]. To elucidate the structures of these products, high resolution mass spectral data [HREI-MS (Jeol DX505W; Jeol, Tokyo, Japan) or HRAPCI-MS (Jeol JMS-T100LP)], and nuclear magnetic resonance (NMR) spectral data (400 MHz, Bruker AMX400) were applied.

## Competing interests

The authors declare that they have no competing interests.

## Authors’ contributions

GS cultured the cyanobacterial strains and prepared genome. TM and HH isolated P450 genes and constructed plasmids for functionally expressing them. TM also carried out screening and bioconversion experiments, and HPLC analyses. TO selected substrates for the screening and participated in the screening and bioconversion experiments and interpretation of the screening results. KS and EK purified the products and did their spectroscopic analyses. NM made substantial contributions to conception and design of experiments and participated in writing the manuscript. All authors read the manuscript and gave final approval of the version to be published.

## Supplementary Material

Additional file 1 **Figure S1. Composition of four distinct P450 monooxygenase systems. Figure S2: Structure of the pRED vector for the functional expression of class I P450 genes in*E. coli.*Figure S3: List of screened substrates (47 samples). Figure S4: CO difference spectral analysis of CYP110E1 C-terminally fused to RhFRed.** Cell extracts from *E. coli* BL21 (DE3) carrying plasmid pCYP110E1-Red (three samples, S1, S2, and S3) were measured for CO difference spectra. (PPT 424 kb) Click here for file

Additional file 2 **Spectroscopic data of the individual converted products.** (DOC 48 kb) Click here for file
