# Supplementary material for: Biocatalytic synthesis of flavones and hydroxyl-small molecules by recombinant Escherichia coli cells expressing the cyanobacterial CYP110E1 gene
Source: Microb Cell Fact. 2012 Jul 18;11:95. doi: 10.1186/1475-2859-11-95 (PMC3411444; doi:10.1186/1475-2859-11-95)
Supplement: Additional file 1 — Figure S1. Composition of four distinct P450 monooxygenase systems. Figure S2: Structure of the pRED vector for the functional expression of class I P450 genes inE. coli.Figure S3: List of screened substrates (47 samples). Figure S4: CO difference spectral analysis of CYP110E1 C-terminally fused to RhFRed. Cell extracts from E. coli BL21 (DE3) carrying plasmid pCYP110E1-Red (three samples, S1, S2, and S3) were measured for CO difference spectra. (PPT 424 kb) [file 1475-2859-11-95-S1.ppt]

## Slide 1
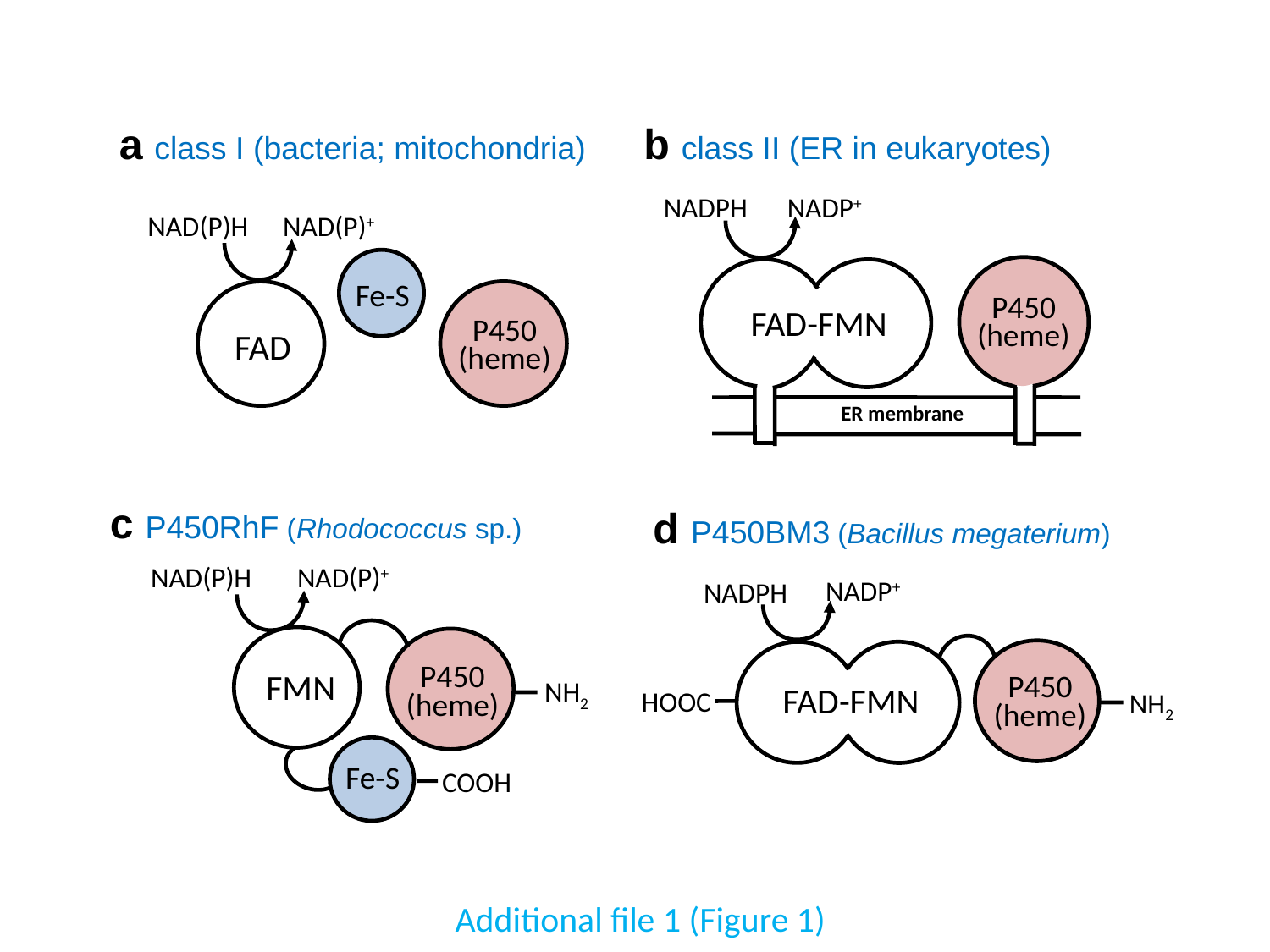

a class I (bacteria; mitochondria)
b class II (ER in eukaryotes)
NADPH
NADP+
P450
(heme)
FAD-FMN
ENDOPLASMIC
RETICULUM
ER membrane
NAD(P)H
NAD(P)+
Fe-S
P450
(heme)
FAD
c P450RhF (Rhodococcus sp.)
d P450BM3 (Bacillus megaterium)
NAD(P)H
NAD(P)+
FMN
P450
(heme)
NH2
Fe-S
COOH
NADP+
NADPH
FAD-FMN
HOOC
NH2
P450
(heme)
Additional file 1 (Figure 1)

## Slide 2
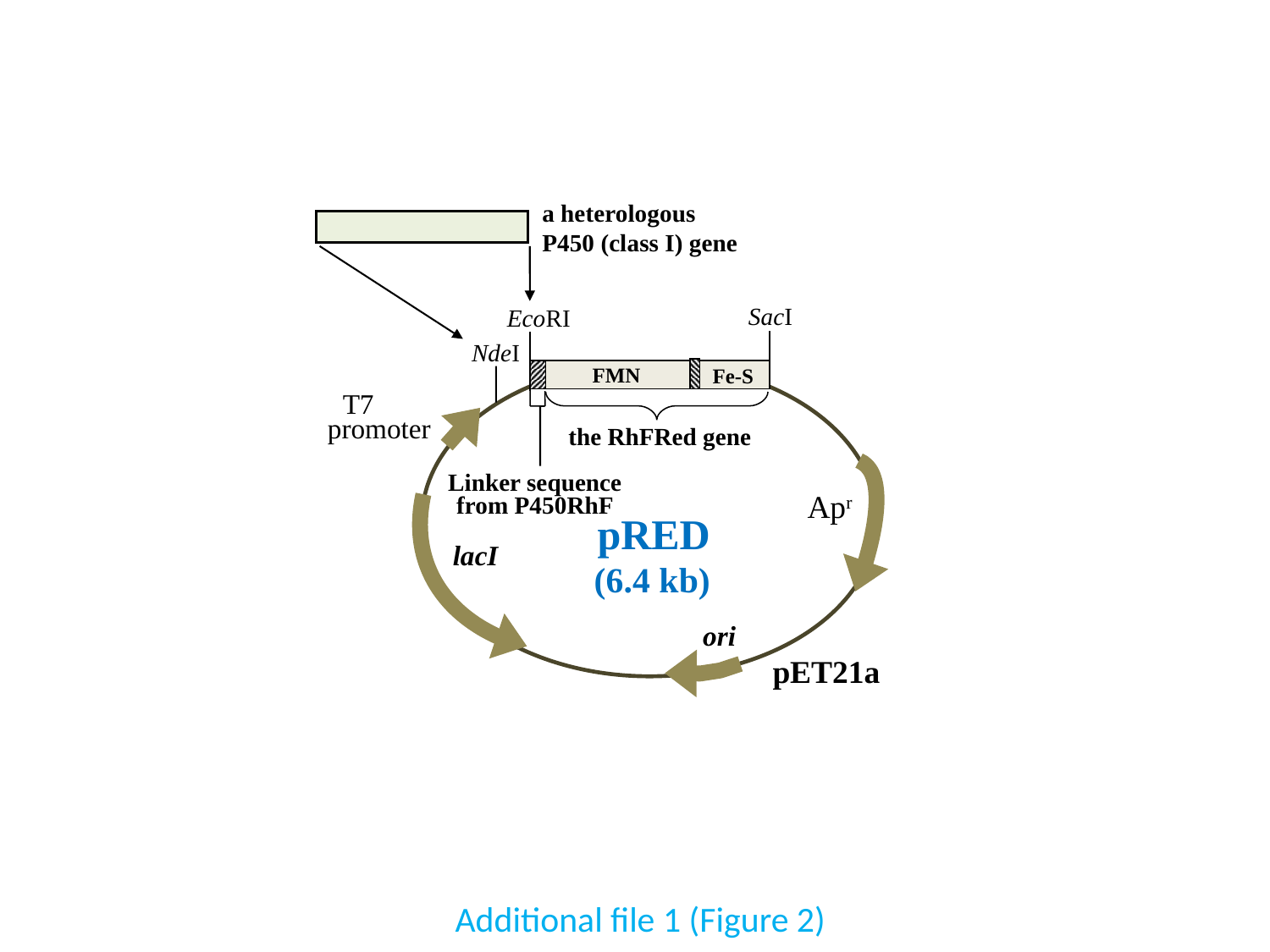

a heterologous P450 (class I) gene
SacI
EcoRI
NdeI
FMN
Fe-S
T7　promoter
the RhFRed gene
Linker sequence
from P450RhF
Apr
pRED
lacI
(6.4 kb)
ori
pET21a
Additional file 1 (Figure 2)

## Slide 3
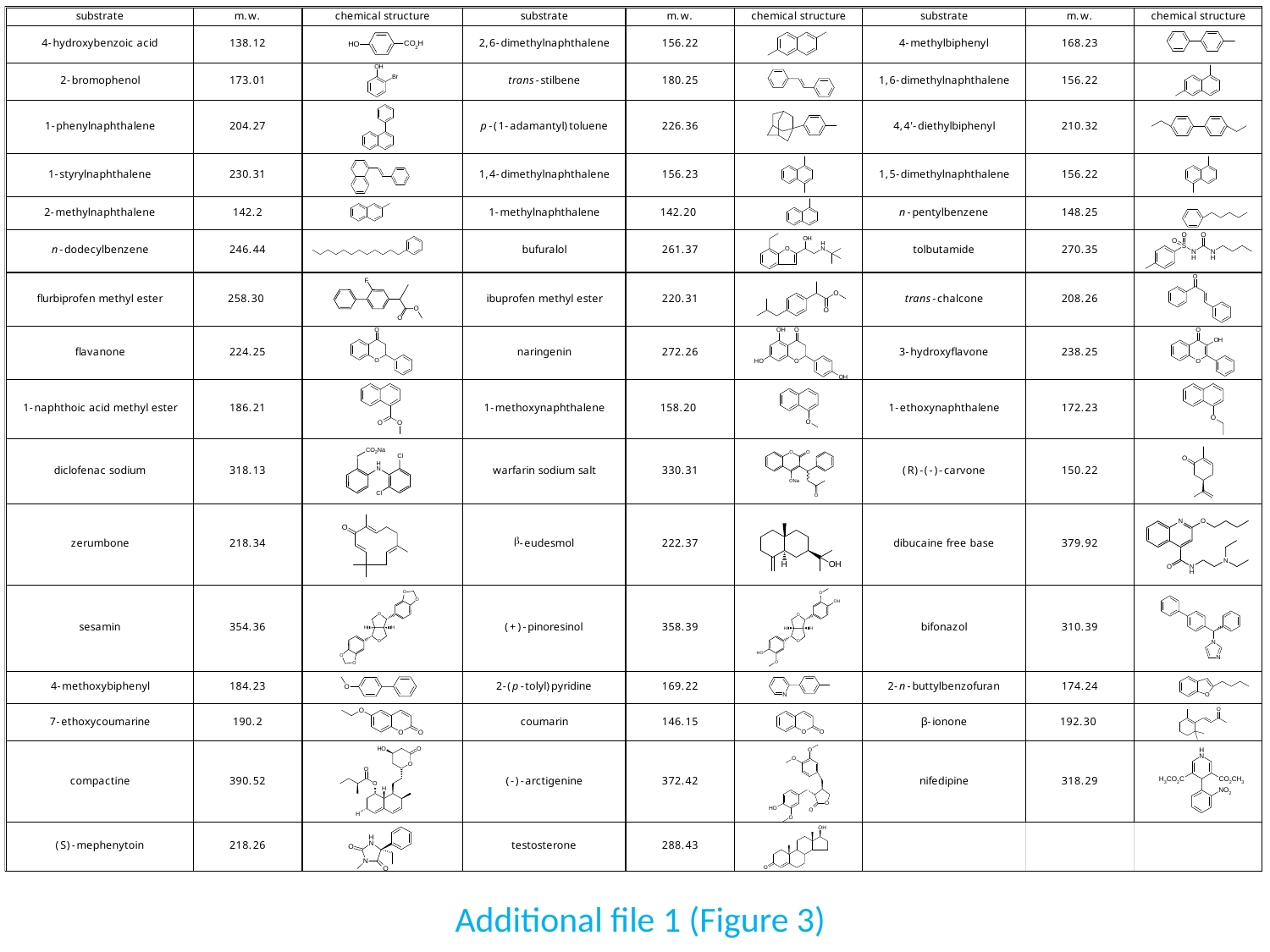

Additional file 1 (Figure 3)

## Slide 4
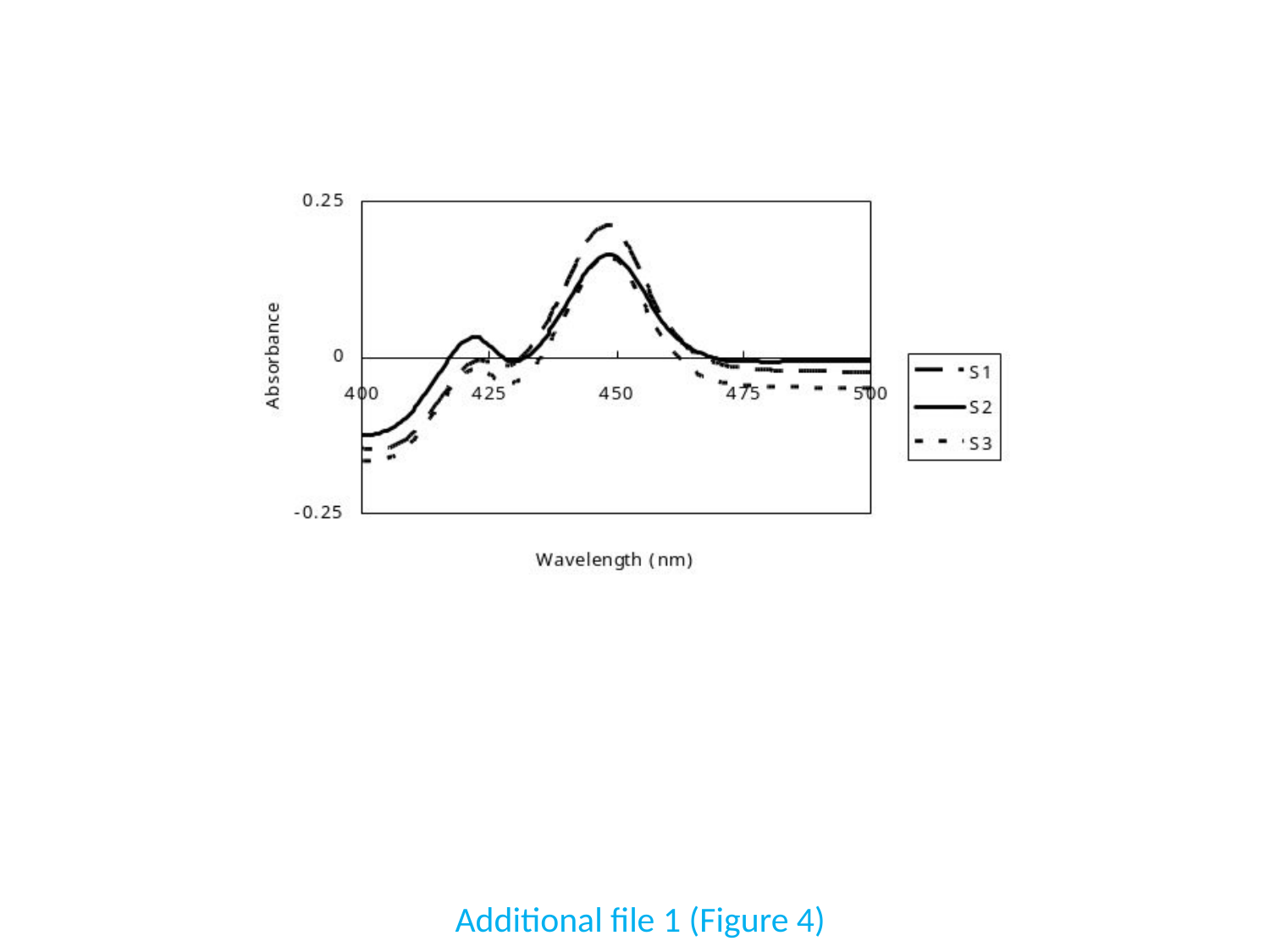

Additional file 1 (Figure 4)
