# Supplementary material for: Biocatalytic synthesis of flavones and hydroxyl-small molecules by recombinant Escherichia coli cells expressing the cyanobacterial CYP110E1 gene
Source: Microb Cell Fact. 2012 Jul 18;11:95. doi: 10.1186/1475-2859-11-95 (PMC3411444; doi:10.1186/1475-2859-11-95)
Supplement: Additional file 2 — Spectroscopic data of the individual converted products. (DOC 48 kb) [file 1475-2859-11-95-S2.doc]

**Additional file 2**

**Spectroscopic data of the individual converted products.**

**S-1** HREI-MS: calculated for C_15_H_24_O (M^+^), 220.1827; found 220.1825. ^1^H-NMR (CDCl_3_) δ: 1.05 (d, *J*=6.8 Hz, H-12), 1.15 (s, 3H, H-14), 1.17 (s, 3H, H-15), 1.25 (m, 1H, H-4b), 1.32 (m, 1H, H-4a), 1.45 (s, 3H, H-13), 1.63-1.68 (2H, H-3), 1.88-1.92 (2H, H-5), 2.06 (d, *J*=8.0 Hz, 2H, H-8), 2.56 (m, 1H, H-2), 5.09 (dd, *J*=8.0, 8.0 Hz, 1H, H-7), 6.05 (d, *J*=16.2 Hz, 1H, H-11), 6.24 (d, *J*=16.2 Hz, H-1, H-10). ^13^C-NMR (CDCl_3_) δ: 14.0 (C-12), 16.5 (C-13), 21.9 (C-4), 25.9 (C-14), 26.3 (C-15), 32.4 (C-3), 40.4 (C-5), 41.6 (C-8), 39.6 (C-9), 47.3 (C-2), 122.2 (C-7), 126.6 (C-11), 137.4 (C-6), 151.9 (C-10), 205.3 (C-1).

**S-2** HREI-MS: calculated for C_15_H_24_O_2_ (M^+^), 236.1776; found 236.1778. ^1^H-NMR (CDCl_3_) δ: 1.03 (d, *J*=6.8 Hz, 3H, H-12), 1.15 (s, 3H, H-14), 1.17 (s, 3H, H-15), 1.18 (m, 1H, H-4b), 1.31 (m, 1H, H-4a), 1.63-1.68 (2H, H-3), 1.91 (m, 1H, H-5b), 2.06 (m, 1H, H-5a), 2.13-2.17 (2H, H-8), 2.56 (m. 1H, H-2), 3.92 (d, *J*=12.2 Hz, 1H, H-13b), 4.02 (d, *J*=12.2 Hz, 1H, H-13a), 5.20 (dd, *J*=8.5, 8.5 Hz, 1H, H-7), 6.04 (d, *J*=16.2 Hz, H-11), 6.12 (d, *J*=16.2 Hz, H-10). ^13^C-NMR (CDCl_3_) δ: 13.8 (C-12), 22.4 (C-4), 25.7 (C-14), 26.4 (C-15), 31.9 (C-3), 35.0 (C-5), 40.9 (C-8), 41.1 (C-9), 47.1 (C-2), 59.5 (C-13), 126.0 (C-7), 126.9 (C-11), 140.1 (C-6), 152.3 (C-10), 205.2 (C-1).

**S-3** HREI-MS: calculated for C_15_H_24_O_2_ (M^+^), 236.1776; found 236.1779. ^1^H-NMR (CDCl_3_) δ: 1.00 (d, *J*=7.0 Hz, 3H, H-12), 1.12 (s, 3H, H-14), 1.20-1.28 (2H, H-4), 1.26 (s, 3H, H-15), 1.50 (s, 3H, H-13), 1.63-1.68 (2H, H-3), 1.90-2.05 (2H, H-5), 2.67 (m, 1H, H-2), 4.24 (d, *J*=9.8 Hz, 1H, H-8), 5.09 (d, *J*=9.8 Hz, 1H, H-7), 6.11 (d, *J*=16.5 Hz, 1H, H-11), 6.20 (d, *J*=16.5 Hz, 1H, H-10). ^13^C-NMR (CDCl_3_) δ: 12.0 (C-12), 16.5 (C-13), 21.8 (C-4), 25.7 (C-14), 25.9 (C-15), 31.2 (C-3), 39.7 (C-5), 43.2 (C-9), 45.7 (C-2), 75.5 (C-8), 125.3 (C-7), 129.0 (C-11), 139.0 (C-6), 149.2 (C-10), 204.7 (C-1).

**A-1** HRAPCI-MS: calculated for C_22_H_17_O_4_ (M-H)^-^, 345.11269; found 345.11245. ^1^H-NMR (CDCl_3_) δ: 4.02 (s, 6H, H-9, H-9’), 6.92 (s, 2H, H-2, H-2’), 7.49 (m, 2H, H-7, H-7’), 7.54 (m, 2H, H-6, H-6’), 8.20 (d, *J*=7.5 Hz, 2H, H-8, H-8’), 8.30 (d, *J*=7.5 Hz, 2H, H-H-5, H-5’). ^13^C-NMR (CDCl_3_) δ: 56.3 (C-9, C-9’), 107.9 (C-2, C-2’), 120.4 (C-3, C-3’), 122.7 (C-8, C-8’), 123.5 (C-5, C-5’), 126.2 (C-8a, C-8a’), 126.5 (C-7, C-7’), 127.0 (C-6, C-6’), 128.5 (C-4a, C-4’a), 142.1 (C-4, C-4’), 149.3 (C-1, C-1’).

**A-2** HRAPCI-MS: calculated for C_11_H_9_O_2_ (M-H)^-^, 173.18808; found 173.18822. ^1^H-NMR (CDCl_3_) δ: 3.99 (s, 3H, H-9), 6.84 (d, *J*=7.8 Hz, H-2), 6.84 (d, *J*=7.8 Hz, H-6), 7.30 (dd, *J*=7.8, 8.4 Hz, H-7), 7.39 (dd, *J*=7.8, 8.6 Hz, H-3), 7.74 (d, *J*=8.6 Hz, H-4), 7.85 (d, *J*=8.4 Hz, H-8). ^13^C-NMR (CDCl_3_) δ: 55.6 (C-9), 104.5 (C-2), 109.5 (C-6), 113.6 (C-4), 114.7 (C-8), 125.1 (C-7), 125.3 (C-4a), 125.3 (C-3), 126.9 (C-8a), 151.2 (C-5), 155.4 (C-1).

**A-3** HRAPCI-MS: calculated for C_11_H_9_O_2_ (M-H)^-^, 173.18808; found 173.18831. ^1^H-NMR (CDCl_3_) δ: 3.98 (s, 3H, H-9), 5.77 (s, 1H, OH-2), 7.23 (d, *J*=8.5 Hz, 1H, H-3), 7.36 (dd, *J*=8.0, 8.4 Hz, 1H, H-6), 7.49 (dd, *J*=8.0, 8.2 Hz, 1H, H-7), 7.57 (d, *J*=8.5 Hz, 1H, H-4), 7.79 (d, *J*=8.0 Hz, 1H, H-5), 7.94 (d, *J*=8.2 Hz, 1H, H-8). ^13^C-NMR (CDCl_3_) δ: 61.6 (C-9), 117.4 (C-3), 120.3 (C-8), 123.5 (C-6), 125.3 (C-4), 126.3 (C-7), 128.0 (C-8a), 128.3 (C-5), 139.2 (C-4a), 144.3 (C-1), 145.4 (C-2).

**A-4** HRAPCI-MS: calculated for C_12_H_11_O_2_ (M-H)^-^, 187.07591; found 187.07565. ^1^H-NMR (CDCl_3_) δ: 1.52 (t, *J*=7.0 Hz, 3H, H-10), 4.16 (q, *J*=7.0 Hz, 2H, H-9), 7.23 (d, *J*=8.7 Hz, 1H, H-3), 7.33 (dd, *J*=7.9, 8.1 Hz, 1H, H-6), 7.47 (dd, *J*=8.1, 8.6 Hz, 1H, H-7), 7.55 (d, *J*=8.7 Hz, 1H, H-2), 7.78 (d, *J*=7.9 Hz, 1H, H-5), 7.92 (d, *J*=8.6 Hz, 1H, H-8). ^13^C-NMR (CDCl_3_) δ: 15.9 (C-10), 70.1 (C-9), 117.3 (C-3), 120.5 (C-8), 123.5 (C-6), 125.1 (C-2), 126.2 (C-7), 128.3 (C-5), 128.3 (C-8a), 129.6 (C-4a), 138.5 (C-1), 145.8 (C-4).

**A-5** HRAPCI-MS: calculated for C_12_H_11_O_2_ (M-H)^-^, 187.07591; found 187.07588. ^1^H-NMR (CDCl_3_) δ: 1.54 (t, *J*=7.0 Hz, 3H, H-10), 4.20 (q, *J*=7.0 Hz, 2H, H-9), 6.83 (d, *J*=7.0 Hz, 1H, H-2), 6.85 (d, *J*=6.4 Hz, 1H, H-6), 7.29 (dd, *J*=6.4, 8.6 Hz, 1H, H-7), 7.37 (dd, *J*=7.0, 8.5 Hz, 1H, H-3), 7.71 (d, *J*=8.5 Hz, 1H, H-4), 7.89 (d, *J*=8.6 Hz, 1H, H-8). ^13^C-NMR (CDCl_3_) δ: 14.9 (C-10), 63.8 (C-9), 105.3 (C-2), 109.4 (C-6), 113.4 (C-4), 114.9 (C-8), 125.0 (C-7), 125.3 (C-3), 125.4 (C-4a), 127.3 (C-8a), 151.2 (C-5), 154.8 (C-1).

**A-6** HRAPCI-MS: calculated for C_11_H_9_O (M-H)^-^, 157.06534; found 157.06531. ^1^H-NMR (CDCl_3_) δ: 2.45 (s, 3H, H-9), 6.67 (s, 1H, H-3), 7.22 (s, 1H, H-1), 7.40 (dd, *J*=8.0, 8.0 Hz, 1H, H-6), 7.43 (dd, *J*=8.0, 8.6 Hz, 1H, H-8), 8.10 (d, *J*=8.0 Hz, 1H, H-5). ^13^C-NMR (CDCl_3_) δ: 21.8 (C-9), 110.8 (C-3), 119.8 (C-1), 121.3 (C-5), 122.6 (C-4a), 124.3 (C-7), 126.5 (C-6), 127.0 (C-8), 134.9 (C-8a), 135.8 (C-2), 149.2 (C-4).

**A-8** HRAPCI-MS: calculated for C_12_H_11_O (M-H)^-^, 171.08100; found 171.08121. ^1^H-NMR (CDCl_3_) δ: 2.54 (s, 3H, H-10), 2.58 (s, 3H, H-9), 6.70 (d, *J*=7.9 Hz, 1H, H-3), 7.05 (d, *J*=7.9 Hz, 1H, H-2), 7.37 (d, *J*=8.6 Hz, 1H, H-7), 7.84 (d, *J*=8.6 Hz, 1H, H-8), 7.97 (s, 1H, H-5), ^13^C-NMR (CDCl_3_) δ: 18.8 (C-9), 21.7 (C-10), 108.2 (C-3), 124.1 (C-8), 125.1 (C-2), 126.5 (C-4a), 126.6 (C-1), 128.4 (C-7), 131.7 (C-8a), 134.5 (C-6), 151.0 (C-4).

**A-13** HREI-MS: calculated for C_12_H_11_NO M^+^ 185.0841, found 185.0843. ^1^H-NMR (CDCl_3_) δ: 4.77 (s, 2H, H-7), 7.24 (m, 1H, H-5’), 7.48 (d, J=8.1 Hz, 2H, H-3, H-5), 7.99 (d, *J*=8.1 Hz, 2H, H-2, H-6), 7.75 (m, 1H, H-3’), 7.76 (m, 1H, H-4’), 8.70 (d, *J*=4.2 Hz, 1H, H-6’). ^13^C-NMR (CDCl_3_) δ: 65.1 (C-7), 120.6 (C-3’), 122.1 (C-5’), 127.1 (C-6), 127.3 (C-3, C-5), 138.8 (C-1), 138.8 (C-4’), 141.2 (C-4), 157.1 (C-2’), 157.2 (C-6’).

**A-14** HREI-MS: calculated for C_14_H_20_O_3_ M^+^, 236.1412; found 236.1422. ^1^H-NMR (CD_3_Cl) δ: 1.23 (s, 6H, H-12, H-13), 1.50 (d, *J*=7.0 Hz, 3H, H-3), 2.74 (s, 2H, H-10), 3.67 (s, 3H, H-14), 3.72 (q, *J*=7.0 Hz, 1H, H-2), 7.17 (d, *J*=8.2 Hz, 2H, H-6, H-8), 7.24 (d, *J*=8.2 Hz, 2H, H-5, H-9). ^13^C-NMR (CD_3_Cl) δ: 18.6 (C-3), 29.2 (C-12, C-13), 45.1 (C-2), 49.4 (C-10), 52.0 (C-14), 70.7 (C-11), 127.3 (C-5, C-9), 130.8 (C-6, C-8), 136.6 (C-7), 138.8 (C-4), 175.1 (C-1).

**A-15** HREI-MS: calculated for C_16_H_15_FO_3_ M+, 274.1005; found 274.1007. ^1^H-NMR (CD_3_Cl) δ: 1.80 (s, 3H, H-3), 3.83 (s, 3H, H-16), 7.37 (m, 1H, H-13), 7.40 (m, 1H, H-5), 7.40 (d, *J*=7.3 Hz, 1H, H-9), 7.43 (m, 2H, H-12, H-14), 7.45 (m, 1H, H-8), 7.54 (d, *J*=7.5 Hz, 2H, H-11, H-15) . ^13^C-NMR (CD_3_Cl) δ: 26.9 (C-3), 53.5 (C-16), 74.7 (C-2), 113.0 (C-5), 121.2 (C-9), 126.9 (C-7), 127.8 (C-13), 128.4 (C-12, C-14), 128.9 (C-11, C-15), 130.6 (C-8), 134.6 (C-10), 143.6 (C-4), 158.5 (C-6), 175.3 (C-1).

**A-16** HRAPCI-MS: calculated for C_16_H_14_FO_3_ (M-H)^-^, 273.0927; found 273.0911. ^1^H-NMR (CD_3_Cl) δ: 1.53 (d, *J*=7.4 Hz, 3H, H-3), 3.70 (s, 3H, H-16), 3.75 (q, *J*=7.4 Hz, 1H, H-2), 6.89 (d, *J*=8.6 Hz, 2H, H-12, H-14), 7.09 (dd, *J*=1.6, 10.5 Hz, 1H, H-5), 7.11 (dd, *J*=1.5, 7.6 Hz, 1H, H-9), 7.34 (dd, *J*=7.6, 8.8 Hz, 1H, H-8), 7.41 (dd, *J*=1.5, 8.8 Hz, 2H, H-11, H-15). ^13^C-NMR (CD_3_Cl) δ: 18.4 (C-3), 44.9 (C-2), 52.2 (C-16), 115.3 (C-5), 115.4 (C-12, C-14), 123.5 (C-9), 127.5 (C-7), 128.0 (C-10), 130.3 (C-11, C-15), 130.5 (C-8), 141.2 (C-4), 155.3 (C-13), 159.7 (C-6), 174.6 (C-1).
